# Evidence based guidelines for complex regional pain syndrome type 1

**DOI:** 10.1186/1471-2377-10-20

**Published:** 2010-03-31

**Authors:** Roberto S Perez, Paul E Zollinger, Pieter U Dijkstra, Ilona L Thomassen-Hilgersom, Wouter W Zuurmond, Kitty CJ Rosenbrand, Jan H Geertzen, the CRPS I task force

**Affiliations:** 1 VU University Medical Center, dept of Anaesthesiology, Amsterdam, the Netherlands; 2 Research consortium Trauma Related Neuronal Dysfunction (TREND), the Netherlands; 3 The EMGO Institute for Health and Care Research, Amsterdam, the Netherlands; 4 Rivierenland Hospital, dept of Orthopaedic Surgery, Tiel, the Netherlands; 5 University Medical Centre Groningen, Center for Rehabilitation, University of Groningen, Groningen, the Netherlands; 6 Dutch Association of Posttraumatic Dystrophy Patients, Nijmegen, the Netherlands; 7 Dutch Institute for Healthcare Improvement CBO, Utrecht, the Netherlands

## Abstract

**Background:**

Treatment of complex regional pain syndrome type I (CRPS-I) is subject to discussion. The purpose of this study was to develop multidisciplinary guidelines for treatment of CRPS-I.

**Method:**

A multidisciplinary task force graded literature evaluating treatment effects for CRPS-I according to their strength of evidence, published between 1980 to June 2005. Treatment recommendations based on the literature findings were formulated and formally approved by all Dutch professional associations involved in CRPS-I treatment.

**Results:**

For pain treatment, the WHO analgesic ladder is advised with the exception of strong opioids. For neuropathic pain, anticonvulsants and tricyclic antidepressants may be considered. For inflammatory symptoms, free-radical scavengers (dimethylsulphoxide or acetylcysteine) are advised. To promote peripheral blood flow, vasodilatory medication may be considered. Percutaneous sympathetic blockades may be used to increase blood flow in case vasodilatory medication has insufficient effect. To decrease functional limitations, standardised physiotherapy and occupational therapy are advised. To prevent the occurrence of CRPS-I after wrist fractures, vitamin C is recommended. Adequate perioperative analgesia, limitation of operating time, limited use of tourniquet, and use of regional anaesthetic techniques are recommended for secondary prevention of CRPS-I.

**Conclusions:**

Based on the literature identified and the extent of evidence found for therapeutic interventions for CRPS-I, we conclude that further research is needed into each of the therapeutic modalities discussed in the guidelines.

## Background

Complex Regional Pain Syndrome type I (CRPS-I) is a condition that causes multiple problems for both patients and practitioners, due to the large variety of available treatment options. The IASP (International Association for the Study of Pain) definition of the syndrome reads as follows: *"CRPS Type I is a syndrome that usually develops after an initiating noxious event, is not limited to the distribution of a single peripheral nerve, and is apparently disproportioned to the inciting event. It is associated at some point with evidence of oedema, changes in skin blood flow, abnormal sudomotor activity in the region of the pain, or allodynia or hyperalgesia" *[1].

A distinction was made between CRPS type I, formerly known as reflex sympathetic dystrophy (RSD), and type II, where a nerve lesion can be detected (formerly known as causalgia) [1,2].

The condition often starts in an arm or leg, and is characterized by a combination of autonomic, sensory and vasomotor symptoms. Pain, temperature asymmetry impaired movement, change in skin colour, hyperaesthesia, hyperalgesia, hyperpathy, tremor, involuntary movements, muscle spasms, paresis, pseudoparalysis, skin, muscle and bone atrophy, hyperhidrosis and changes in hair and nail growth have been reported in patients with this syndrome [3]. It usually requires long-term, intensive medical therapy whereby many CRPS-I patients are no longer able to perform their usual (social) role in everyday life. As a result, CRPS-I has a major impact on quality of life [4,5].

Various sets of diagnostic criteria are used side by side, and many different therapies have been applied to this patient group. The complexity of this problem, the fact that various disciplines are involved in treatment, and the consequences for the patient's psychosocial functioning mean that a clear, uniform set of guidelines is essential. In the light of the foregoing considerations, a multidisciplinary task force was instigated by the Dutch Society of Rehabilitation Specialists and the Dutch Society of Anaesthesiologists in order to draw up evidence-based guidelines for CRPS-I treatment.

## Methods

A multidisciplinary task force was set up in the autumn of 2003. The task force included representatives of all medical and paramedical disciplines engaged in diagnosing and treating patients with CRPS-I, epidemiologists, a representative of the Dutch Association of Posttraumatic Dystrophy Patients and the Dutch Institute for Healthcare Improvement CBO. An even spread between geographical locations, balanced representation of the various societies and bodies involved, and a fair division between members with an academic background and those from a non-academic background was ensured.

Members of the task force were further subdivided into project groups addressing specific areas of CRPS treatment. Relevant articles written in English, German, French, Italian or Dutch were identified by searches in the Cochrane Library, Medline, Embase, Cinahl and Psychinfo, using a search string based on the PICO method (see additional file 1). Reference lists of articles identified were screened for relevant articles that did not come up in the database search, and recent guidelines were consulted. Studies were selected based on their methodological strength (meta-analyses, systematic reviews, randomized controlled trials (RCT's) and controlled trials (CT's)). Systematic reviews and meta-analyses, considered to have the highest evidential strength, were given precedence over individual articles included in the review. In case these studies were not available, comparative cohort studies, comparative patient control trials or non-comparative trials were used in the evaluations. Other important criteria were: adequate size, adequate follow-up, adequate exclusion of selection bias, and whether the results obtained can be generalized to the Dutch health care system. The search covered the period from 1980 to June 2005, although some studies published prior to or after this period were also used for the guidelines.

The members of the project group assessed the quality of these studies on the basis of evidence-based guideline development (EBGD) assessment forms. The studies were then graded according to their strength of evidence as described in table 1. Conclusions were formulated based on the available strength of evidence, and described in terms in accordance with the levels shown in table 1. These descriptions ranged from clear statements about efficacy ("It is proven that...") for level one down to expressions of expert opinion ("The task force is of the opinion that...") for level four conclusions.

**Table 1:** Classification of the literature consulted, according to strength of evidence

Level of evidence for studies on intervention:	
A1	systematic reviews that comprise at least several A2 quality trials whose results are consistent
A2	high-quality randomized comparative clinical trials (randomized, double-blind controlled trials) of sufficient size and consistency
B	randomized clinical trials of moderate quality or insufficient size, or other comparative trials (non-randomized, comparative cohort study, patient control study)
C	non-comparative trials
D	opinions of experts, such as project group members

**Level of evidence for conclusions:**	

1	at least one systematic review (A1) or two independent grade A2 studies
2	at least two independent grade B studies
3	at least one grade A2, B or C study
4	opinions of experts, such as project group members

The project group members produced texts, either individually or in subgroups. These texts comprised background information with respect to the intervention and studies assessed, conclusions regarding the efficacy of the intervention according to strength of evidence and additional considerations concerning treatment related issues (i.e. availability of treatment methods, side effects, cost-effect benefits, consequences for organization of care etc.). All texts including recommendation were discussed at plenary meetings and approved after comments had been taken into account. The plenary project group met ten times to discuss draft texts. The draft guidelines were sent for external review to the participating professional societies (see acknowledgements for details) and were presented and discussed at an open national meeting. Once all comments had been taken into account, the guidelines were adopted by the full project group and sent to the participating professional organisations for final approval. Formal endorsement was obtained in December 2006.

The conclusions for each treatment modality including strength of evidence and underlying literature will be presented in italics in the result section. The final recommendations for the different treatment modalities will be described at the end of this article. A practical algorithm based on these guidelines is presented in additional file 2.

## Results

Database and cited reference search revealed 94 relevant studies after selection. These included 25 studies on oral or topical drug interventions, 42 studies on invasive treatments, 15 on paramedical interventions, 4 on primary and 8 on secondary prevention of CRPS. Treatment interventions for children with CRPS, comprising 8 studies were described separately.

### Drug treatment

#### Pain medication

Although analgesics are often used when treating patients with CRPS-I, and their use is described in various treatment protocols and guidelines [6-8], the scientific support for their administration to patients with CRPS-I is very limited.

#### Paracetamol

The use of paracetamol is described in the context of an adjuvant pain protocol in a study into the efficacy of free radical scavengers in treating CRPS-I (n = 146) [9]. No studies were found evaluating paracetamol as a stand-alone treatment for CRPS-I.

*There is no evidence that paracetamol is effective in treating pain in CRPS-I patients (level 4)*.

#### NSAIDs

Sixty-one CRPS-I patients were retrospectively evaluated with respect to the effects of 60 mg of keterolac administered by means of a regional intravenous blockade [10]. Twenty-six percent of patients had a complete response, 42% had a partial response and 31% had no response. Patients with allodynia had significantly less response to the treatment. Conflicting data have been published with regard to the use of NSAIDs in patients with neuropathic pain [11].

*There is insufficient evidence of the degree of pain control achieved by NSAIDs in CRPS-I patients (level 3:Connelly et al. (C))*.

#### Opioids

One placebo-controlled RCT (n = 43) was found investigating the effects of sustained-release oral morphine on patients who had previously been treated with epidural spinal cord electrical stimulation (ESES) [12]. No significant differences were found between the extent of pain reduction and the average time for ESES to become effective. On average, the morphine group reported 20 side-effects a day, against 2 a day in the placebo group. An uncontrolled study with 9 CRPS I and II patients evaluated the effects of continuous infusion of morphine in the axillary plexus following stellate blockade [13]. Significant pain reduction at rest and at movement and increased grip strength were found. However, the steady-state morphine concentrations were lower than the minimally effective analgesic concentration. There is little information on weak and strong acting opioids in patients with CRPS-I. Systematic reviews on their use for neuropathic pain have found tramadol to be effective [14]. Positive short-term effects have also been reported for strong acting opioids administered for neuropathic and musculoskeletal pain [15].

*There is insufficient evidence for the effects of oral opioids in CRPS-I patients on pain (level 3: Harke et al. (B))*.

*There is insufficient evidence for the effects of infusion of morphine to the axillary plexus on pain in CRPS-I patients (level 3: Azad et al. (C))*.

#### Local anaesthetics

One quasi-experimental study with 7 patients evaluated the effects of lumbar and stellate blockades with lidocaine/bupivacaine compared to placebo (follow up 2-2,5 weeks). No significant differences were found between active and placebo treatment with respect to initial peak pain reduction [16].

An uncontrolled open study investigated the long term effects (mean follow up 32 months, range 7-48 months) of epidural administration of bupivacaine to 14 patients with CRPS-I in the knee [17]. Treatment was continued with continuous administration of a narcotic. No pain control data were described, however, 11 patients were seen to have a complete improvement of CRPS-I symptoms at the end of the follow-up.

*There is insufficient evidence to allow any statement about the efficacy of local anaesthetics administered to the sympathetic ganglia in CRPS-I patients (level 3: Price et al. (B), Azad et al (C))*.

*There is insufficient evidence to allow any statement about the efficacy of epidural administered local anaesthetic to CRPS-I patients. Due to use of different interventions the efficacy of epidural administration of local anaesthetics cannot be determined (level 3: Cooper et al. (C))*.

#### Anaesthetics

The effects of a sub-anaesthetic ketamine infusion (10 mg/hour up to 15-50 mg/hour) was assessed in a retrospective study of 33 patients with CRPS-I or -II [18]. Twelve patients experienced a relapse and had a second course of infusions, three patients had a third course, by which pain disappeared completely in 83% of patients. The average duration of pain reduction (data of 20 patients) was 9.4 months. The side-effects were intoxication, hallucinations, dizziness, nausea, light-headedness and blurred vision.

*There are indications that intravenous administration of a sub-anaesthetic dose of ketamine reduces pain in CRPS-I patients (level 3: Correll et al. (C))*.

#### Anticonvulsants

Two placebo-controlled, randomized studies have been found that examined the use of gabapentin in neuropathic pain patients. The first study [19] (n = 307) shows that gabapentin causes a modest but significant reduction in neuropathic pain symptoms eight weeks after the start of treatment. It is unclear what this means for the CRPS-I patients, who made up 28% of the sample population.

In the second study (n = 58) a moderate effect on pain was found, but no significant reductions in other sensory abnormalities were found [20]. Dizziness, sleepiness and fatigue occurred significantly more often in patients taking gabapentin than in patients taking placebo.

*There are indications that gabapentin administered at doses of 600 to 1800 mg every 24 hours in the first eight weeks can cause some reduction in pain symptoms suffered by patients with CRPS-I*.

*There is limited evidence that gabapentin reduces sensory abnormalities such as hyperaesthesia and allodynia. The long-term effect of gabapentin on patients with CRPS-I is not known (level 2: Serpell (B), Van de Vusse et al. (B))*.

No studies were found evaluating the effects for other anticonvulsants in relation to CRPS I.

*There is no evidence that anticonvulsants such as carbamazepine, pregabalin and phenytoin are effective in reducing pain in CRPS-I patients (level 4)*.

#### Antidepressants

No studies testing tricyclic antidepressants on patients with CRPS-I were available.

*There is no evidence that antidepressants are effective in reducing pain in patients with CRPS-I (level 4)*.

#### Capsaicin

Only one study has been found in which an extremely high dose of capsaicin (5 to 10%) was administered to ten patients with CRPS-I [21]. Doses of this strength can only be spread onto patients' skin if the painful body part is first numbed by epidural anaesthesia.

The investigators claim to have succeeded in 90% of patients in achieving pain reduction. No scientific conclusions can yet be drawn from this open-label study.

There is insufficient evidence that capsaicin is effective in CRPS-I patients (level 4)

#### Free radical scavengers

A prospective crossover study [22] with 20 patients found a positive effect of dimethylsulphoxide (DMSO) on the function of the affected limb. In 26 CRPS-I patients DMSO was found to be significantly more effective than the conventional regional ismelin block [23] in reducing pain. A randomised double-blind trial conducted with 32 CRPS-I patients [24] showed that 5 times daily use of DMSO in cremor vaselini cetomacrogolis provided significantly better results on CRPS-I symptoms than placebo after two months of treatment. A randomized double-blind study in 146 CRPS-I patients found comparable results for DMSO cream and N-acetylcysteine [9]. In general, DMSO generates lower (direct and indirect) costs than N-acetylcysteine. Subgroup analysis indicates that N-acetylcysteine is more cost effective in patients with a cold form of CRPS-I than DMSO. The opposite holds for warm forms of CRPS-I [25].

*DMSO (dimethylsulphoxide) cream (50%) reduces the symptoms of CRPS-I patients (level 2: Perez et al (A2), Geertzen et al. (B); Goris et al. (B), Zuurmond et al. (B))*.

*It is likely that 600 mg of N-acetylcysteine administered three times a day reduces the symptoms of CRPS-I (level 3: Perez et al. (A2))*.

*There are indications that 50% DMSO (dimethylsulphoxide) cream is more effective on primary warm CRPS-I while N-acetylcysteine (NAC) is more effective on primary cold CRPS-I (level 3: Perez et al. (C))*.

#### Oral muscle relaxants

Motor symptoms of CRPS-I may include paresis, dystonia, myoclonias and/or tremor. Five descriptive studies have been conducted into movement disorders in CRPS-I patients (n = 5-43) [26-30]. Three of these studies reported that a small number of CRPS-I patients with dystonia/spasms did benefit from treatment with benzodiazepines and high doses of baclofen [28]. No controlled studies have been carried out on the treatment of either dystonia or spasms in patients with CRPS-I. Two descriptive studies report that anticholinergics have never produced (lasting) effects [29,30].

*There is insufficient evidence of the efficacy of muscle relaxants in treating movement disorders associated with CRPS-I, such as dystonia and muscle spasms (level 3: Bathia et al. (C), Van Hilten et al. (C), Jankovic et al. (C), Marsden et al. (C), Schwartzman et al. (C))*.

#### Botulin toxin

One study described the use of botulin toxin A to treat 14 patients with very severe tonic dystonia of the hand ('clenched fist') [31]. In four of these patients, the dystonia developed in the context of CRPS-I. An 'overall' improvement in pain and muscle relaxation was achieved in four out of five hands, but the extent of improvement was not described. Other articles report that botulin toxin injections never work, or only work for a short period, and rarely lead to improvement in functionality [27,29].

*There is insufficient evidence that botulin toxin A is effective in treating dystonia in CRPS-I patients (level 3: Cordivari et al. (C), Van Hilten et al. (C), Jancovic et al. (C))*.

#### Intrathecal baclofen administration

Intrathecal baclofen therapy (ITB) is an invasive technique that has only been investigated in two patients with CRPS-I alone [32] and seven CRPS-I-dystonia patients [33] whose condition had failed to respond to previous treatment. Only the latter study was preceded by a double-blind placebo-controlled crossover screening procedure aimed to ascertain whether patients would be suitable for having a programmable pump for ITB fitted. Comparison with a placebo revealed that baclofen significantly improved outcomes. Six patients underwent the implant procedure and were monitored for 1.7 years as part of an open trial with varying degrees of success. Zuniga et al. also reported an open trial with ITB on two CRPS-I patients with no motor disorder [32]. Pain, allodynia and autonomic disorders responded well to ITB. The main side-effects of the screening process and continuous administration of ITB are post-puncture headache, diminished consciousness and urine retention [33].

*There is insufficient evidence that intrathecal baclofen (ITB) is effective in treating dystonia in CRPS-I patients (level 3: Van Hilten et al. (C), Zuniga et al. (C))*.

#### Corticosteroids

Corticosteroids have been used in open trials (n = 64-69) [34,35] and in one controlled trial (n = 23) [36] to treat CRPS-I, all of limited methodological quality. All the studies found corticosteroids to have a very pronounced beneficial effect.

*Corticosteroids may have a positive effect on CRPS-I. Little is known as to the duration and dosage (level 3: Christensen et al. (C), Grundberg et al. (C), Kozin et al. (C))*.

#### Calcitonin

The effects of calcitonin have been evaluated in two meta-analyses and two systematic reviews. The meta-analysis carried out by Kingery et al. [37] reports conflicting findings as to the effects of calcitonin. The systematic review conducted by Van den Berg et al. [38] finds no evidence that calcitonin is effective in cases of CRPS-I. In contrast, the meta-analysis carried out by Perez et al. points to calcitonin having a positive effect on pain on average [39], and the review carried out by Forouzanfar et al. also describes positive results for calcium-regulating drugs (including calcitonin) administered to CRPS-I patients [40].

*There is conflicting evidence with respect to the efficacy of calcitonin for treatment of CRPS-I (level 1: Van den Berg et al. (A1), Forouzanfar et al. (A1), Kingery (A1), Perez et al. (A1))*.

#### Bisphosphonates

Three placebo-controlled studies have been carried out to date [40-43]. One study (n = 20) involved administration of alendronate three days in a row [41]. Another study evaluated the efficacy of clodronate (n = 32) [42]. In a third study, treatment comprised alendronate (40 mg: this dose is four times as high as that given for osteoporosis) administered to 40 CRPS-I patients [43]. In the three studies, the parameters in the group of patients treated with bisphosphonates improved significantly more than in the placebo group.

*Bisphosphonates have a beneficial effect on the signs of inflammation in patients with CRPS-I. At present little is known as to the optimum dosage, frequency and duration of treatment (level 1: Forouzanfar et al. (A1), Manincourt et al. (A2))*.

### Calcium-channel blockers

Two studies of moderate quality and size investigated the effect of nifedipine and phenoxybenzamine in treating CRPS-I [44,45]. One retrospective study with 59 patients reports that nifedipine (20 mg per day) or phenoxybenzamine (up to 120 mg/day) are most effective for CRPS-I in the acute phase [44]. Both studies are primarily descriptive and the outcomes are subjective, failing to describe the nature of the improvement in patients' conditions.

*There are indications that calcium-channel blockers have some effect in the acute phase of CRPS-I. While they improve blood circulation, they also cause side-effects such as a drop in blood pressure and headache (level 3: Muizelaar et al. (C), Prough et al. (C))*.

### Invasive treatment

#### Intravenous sympathetic blockade

Eight studies have been carried out into the effects of intravenous guanethidine on CRPS-I [23,46-52]. The doses administered ranged from 10 to 30 mg. Four of these studies (n = 9-60) were randomized, comparing guanethidine to a placebo (in most cases 0.9% NaCl) [46,48,50,52]. The remaining studies (n = 20-55) examining the effect of guanethidine report a temporary effect in approximately one-third of patients.

Three additional studies were very small (n = 5-7) from which no conclusions can be drawn [53-55]. One study (n = 16) described a temporary effect of intravenous lidocaine on mechanical and thermal allodynia [56]. Intravenous blockades brought about by guanethidine, lidocaine, clonidine, droperidol and reserpine have been investigated in two meta-analyses and one systematic review [37,39,40], which provided no evidence in favour of intravenous sympathetic blockades.

*Intravenous sympathetic blockade has no added value (pain reduction) compared to placebo in CRPS-I patients (level 1: Kingery (A1), Forouzanfar et al. (A1), Perez et al. (A1))*.

#### Other intravenous treatment

A number of intravenous drugs have been tested for efficacy. Intravenous regional blockades produced by bretylium and ketanserine were found to achieve a significant reduction in pain in the treatment group [57,58]. Ketanserine (n = 16; 10 mg for upper extremity and 20 mg for lower extremtity administration), and two intravenous applications of bretylium at 1.5 mg/kg with lidocaine (in 12 patients) provided slight pain relief. Intravenous administration of reserpine, droperidol and atropine had no effect [37].

*There are indications that 10-20 mg of ketanserine administered by intravenous injection reduces pain in CRPS-I patients. Reserpine, droperidol and atropine do not relieve pain in CRPS-I patients (level 1: Kingery (A1), Hanna et al. (B), Hord et al. (B))*.

#### Percutaneous sympathetic blockade

The literature contains one systematic review of the therapeutic role of local anaesthetic sympathetic blockades in patients with CRPS-I [59]. That review assessed 29 studies performed on 1144 patients with CRPS-I, and concludes that critical examination of the studies raises the question of whether sympathetic blockade is of any benefit at all in CRPS-I. Less than a third of the patients reported temporary relief of pain symptoms following a sympathetic blockade. However, it is unclear whether this is due to a placebo effect.

*Routine administration of percutaneous sympathetic blockade in patients with CRPS-I is not useful (level 2: Cepeda et al. (A1))*.

#### Surgical sympathectomy

The efficacy of surgical sympathectomy was addressed in a systematic review [60], based on analysis of retrospective cohort including 7 to 73 individuals [61-65]. All the studies report a clear reduction in pain due to sympathectomy, whereby the extent of pain relief declines over time. Long term follow up studies (> one year) indicate that the chance of success is greatest if treatment is given within three months after the initial trauma [62,63,65].

*There are indications that surgical sympathectomy can relieve pain in CRPS-I (level 3: AbuRahma et al. (C), Bandyk et al. (C), Bosco Vieira Duarte (C), Mailis et al. (C), Schwartzman et al. (C), Singh et al. (C))*.

#### Spinal cord stimulation

Patients with chronic refractory CRPS-I were randomly allocated to spinal cord stimulation (SCS) plus physiotherapy or physiotherapy alone. Trial stimulation proved successful in 24 of the 36 patients; only these patients underwent a procedure to implant a permanent SCS device. Pain intensity reduced by 2.4 cm on a visual analogue scale after six months in the group receiving spinal cord stimulation plus physiotherapy compared to a group receiving only physiotherapy [66]. At two years follow-up, pain decrease in the SCS group was 2.1 cm more than pain decrease observed in the physiotherapy group [67]. Quality of life improved only in the patients with an implanted system; function remained unchanged. Nine of the 24 patients with an implanted system (38%) experienced complications requiring further surgery within two years [66,67].

Two retrospective cohort studies have investigated effects of SCS on pain relief (n = 23-31) [68,69]. All studies relate to carefully selected patients with refractory CRPS-I. There is no scientific evidence for SCS being effective in non-chronic CRPS-I. Complications requiring further surgery do occur in 25-50% of patients [70].

*Spinal cord stimulation administered to CRPS-I patients who are carefully selected and undergo successful trial stimulation causes long-term pain reduction and improves quality of life, but does not improve function (level 3: Kemler et al. (A2), Calvillo et al. (C), Kemler et al. (C), Kemler et al. (C))*.

#### Amputation

Amputation is sometimes performed with the aim to improve quality of life of CRPS I patients with severe complications, such as threatening sepsis or severe functional impairment.

Two retrospective studies [60,71] evaluating CRPS I patients undergoing amputation were found. One study evaluating seven patients with upper-limb CRPS-I [71], reported three satisfied, two indecisive and two unsatisfied patients. In another study, 34 amputations were carried out on 28 patients [72] due to pain, recurrent infections and functional impairment. Two patients were pain-free; ten infections were adequately controlled, and functional improvement was achieved in nine cases. CRPS-I relapse occurred in 28 cases, but 24 patients remained satisfied with their amputation.

*There is insufficient evidence that amputation positively contributes to the treatment of CRPS-I (level 3: Dielissen et al. (C), Stam et al. (C))*.

### Paramedical interventions

#### Physiotherapy

Published articles often recommend 'physiotherapy' as adjuvant treatment, without specifying exactly what this physiotherapy involves. In general, it is emphasized that functional recovery is essential and forms the key to recovery.

A randomized controlled trial (n = 135) showed that physiotherapy given in addition to medical treatment has a clinically relevant effect on the severity of functional impairments [73,74]. Physiotherapy contributes primarily to quicker reduction of pain, abnormal skin temperature, reduced mobility and oedema. In view of the rapid improvement of disorders it is recommended that physiotherapy should be started at an early stage, or soon after the diagnosis is made [73-75], and may be beneficial for chronic CRPS-I [76,77]. Promising results are reported for Mirror therapy (n = 8-13) in reducing pain [78,79]. Standardized pain-contingent physiotherapy aimed at improving patients' ability to cope with the condition has proven to be effective in reducing CRPS symptoms [73,74,80].

*Physiotherapy for upper-limb CRPS-I is likely to have a beneficial impact on the disorders and on how patients cope with the condition (level 2: Oerlemans et al. (A2), McGabe et al. (B), Fialka et al (C))*.

*There are indications that physiotherapy treatment may be beneficial for chronic CRPS-I (level 3: Moseley (B); Van Wilgen et al. (D))*.

*Physiotherapy should form a part of the standard treatment of CRPS-I (level 4)*.

#### Transcutaneous Electrical Nerve Stimulation (TENS)

Articles of limited methodological quality were found describing beneficial effects of TENS in small groups of CRPS-I (n = 10-11) patients [81,82].

*There is insufficient evidence that TENS is effective in the treatment of CRPS-I (level 4)*.

#### Occupational therapy

We found one RCT evaluating the efficacy of occupational therapy in CRPS-I (n = 135) [74]. Occupational therapy provided in addition to medical treatment had a positive effect on the severity of the functional limitations, and appeared to have a positive impact on activity level [73,74].

*Occupational therapy has a positive effect on functional limitations, and is likely to have a positive effect on the activity level of patients with upper-limb CRPS-I (level 3: Oerlemans et al. (A2))*.

#### Rehabilitation medicine

Though no studies have been carried out to date evaluating the efficacy of integrated and coordinated multidisciplinary interventions for CRPS-I, experts argue for a multidisciplinary approach because of the complex nature of the condition, the possibility of a multifactorial cause, and the varying nature of its progress [6,83].

*There is no evidence that multidisciplinary treatment is beneficial for CRPS-I patients (level 4: Stanton-Hicks et al. (D), Rho et al. (D))*.

#### Psychological treatment

It has been suggested that CRPS-I might be caused or worsened and maintained by non-organic factors [23,84]. We found one RCT (n = 28) evaluating cognitive behavioural therapy in children with CRPS-I [85]. Retrospective cohort surveys or cross-sectional studies with no control group and limited follow-up are common. No scientific publications of psychological treatments administered to adults were found.

### Treatment of children with CRPS I

#### Drug and invasive treatment in children

Little research has been published on specific drug or invasive treatments for children with CRPS-I. Most of the information is limited to descriptions of multimodal treatments [86,87], with the use of analgesics only mentioned in passing.

In a case study of limited quality, 13 children with CRPS-I (9-16 years old) [88] were evaluated to assess the effect of home administration of continuous peripheral nerve blockade (ropivacaine) combined with intensive physiotherapy. The continuous analgesia was assessed as excellent, with the motor block lasting for a limited time (12 hours). The children were able to walk within 24 hours, and none of them showed signs of CRPS-I two months later.

A case study of limited quality [89] examined continuous intravenous infusions of carbacyclin derivatives administered over three days combined with physiotherapy and psychological consultation. All 7 children with CRPS-I (aged between 6 and 11) were reported to be symptom-free after a follow-up period of 30 months on average (range: 25 to 37 months). Repeated infusion was necessary in two cases.

*There is insufficient data to allow any conclusions to be drawn as to the effects of continuous peripheral nerve blockade by means of ropivacaine or continuous intravenous infusion with a carbacyclin derivative in children with CRPS-I (level 3: Dadure et al. (C), Petje et al. (C))*.

#### Physiotherapy for children with CRPS-I

No well-designed trials have been carried out evaluating the effects of physiotherapy modalities in children with CRPS-I. Between 47 and 93% of patients (n = 10-46) are reported to recover after physical therapy [86,90]. Physiotherapy (n = 23) given once a week for six weeks appears to have the same effect as physiotherapy given three times a week for six weeks [85].

The number of children experiencing one or more relapses after treatment ranges from 10 to 48% (n = 10-103) [81,91-93].

*There are indications that physiotherapy is helpful for children with CRPS-I. It is not clear which elements of physiotherapy are effective, as different forms of treatment are combined (level 3: Lee et al. (B), Barbier et al. (C), Kesler et al. (C), Maillard et al. (C), Murray et al. (C), Sherry et al. (C), Wesdock et al. (C), Wilder et al. (C))*.

*There are indications that children with CRPS-I may relapse after receiving physiotherapy (10-48%) (level 3:Lee et al. (B), Barbier et al. (C), Kesler et al. (C), Maillard et al. (C), Murray et al. (C), Sherry et al. (C), Wesdock et al. (C), Wilder et al. (C))*.

#### Occupational therapy of children

An intensive treatment program (n = 23-103), comprising occupational therapy, physiotherapy and hydrotherapy, has been reported to be effective [87,92]. No conclusions can be drawn from the existing literature about children with CRPS-I with regard to the efficacy of occupational therapy.

*There are indications that occupational therapy can be beneficial as part of a multidisciplinary approach to treat children with CRPS-I (level 3: Maillard et al. (C), Sherry et al. (C))*.

#### Psychological treatment of CRPS-I in children

Relaxation therapy and biofeedback (described as cognitive behavioural therapy) in combination with physiotherapy has been evaluated for treatment of children with CRPS-I (n = 23) [85]. Relaxation therapy and biofeedback were reported to reduce both pain symptoms and physical function in 57% of cases (n = 72) [91]. It is not possible to ascertain which of the three treatments contributed most to the effects.

*No conclusions can be drawn as to the effect of cognitive behavioural therapy on children with CRPS-I (level 2: Lee et al. (B), Wilder et al. (B), Sherry et al. (C))*.

### Prevention of CRPS-I

#### Primary prevention

##### Vitamin C

In a randomized double-blind trial (n = 123), patients with a wrist fracture treated with a plaster cast were referred for treatment with vitamin C (500 mg/day for 50 days) or a placebo. Seven percent of patients in the group taking vitamin C developed CRPS-I, as against 22% of patients in the control group (absolute risk reduction 15%, and number needed to treat 7) [94].

In a cohort study with a historic control group (n = 95), patients with wrist fractures treated by surgery were given vitamin C (1000 mg/day for 45 days). Two percent of patients in the group treated with vitamin C developed CPRS-I, compared to 10% in the control group [95].

*It is likely that oral administration of 500 mg of vitamin C per day for 50 days from the date of the injury reduces the incidence of CRPS-I in patients with wrist fractures (level 2: Zollinger et al. (A2), Cazeneuve et al. (B))*.

#### Guanethidine

In a randomized study (n = 71), patients scheduled for surgery for Dupuytren's disease were referred for pre-emptive intravenous guanethidine blockade or a placebo blockade. After eight weeks 13% of the patients taking guanethidine were found to have developed CRPS-I, as against 6% in the control group [96].

*There are no indications that perioperative intravenous guanethidine in patients undergoing fasciectomy for Dupuytren's disease has any effect on the incidence of CRPS-I (level 3: Gschwind et al. (A2))*.

#### Calcitonin

In a double blind randomized study, 91 patients undergoing wrist, knee or foot surgery were treated with for 100 IU of thyrocalcitonin administered subcutaneously (from the day of the operation or the trauma once a day for one week and three times a week for three weeks thereafter) or placebo injections. No significant differences were found between placebo and thyrocalcitonin in reducing the occurrence of CRPS-I [97].

*There are no indications that subcutaneous administration of calcitonin for four weeks from the onset of the trauma or from the date of surgery can prevent patients developing CRPS-I (primary prevention) (level 3: Riou et al. (B))*.

### Secondary prevention

Various interventions or combinations of interventions aimed at preventing relapse of CRPS-I have been described, but little adequate research has been carried out. Relapse rates up to 13% (of 47 patients) have been reported despite combined interventions aimed at preventing relapse of CRPS-I (waiting until the symptoms of CRPS-I had abated, minimizing the use of tourniquet, administering vasodilators to encourage circulation, sympathetic blockades and mannitol) [98]. Six percent of patients with a history of CPRS-I (n = 18) treated with calcitonin (100 IU a day *s.c. *for four weeks) had a relapse of CRPS-I, against 28% of the patients in a historic control group (n = 74) [99]. A retrospective study (n = 50) found that peri-operative stellate ganglion blockade carried out to prevent a relapse of CRPS-I to be unsuccessful in 10% of cases. The relapse rate in an untreated control group was 72% [100].

A retrospective study (n = 1200) found that 1% of the patients undergoing anterior cruciate ligament surgery receiving pre-emptive analgesia (comprising administration of paracetamol and NSAIDs before surgery) combined with multimodal analgesia experienced a relapse of CPRS-I. The CRPS-I relapse rate for a control group, taking painkillers only as required after surgery, was 4% [101].

In a randomized double-blind study in 84 patients with a history of CRPS-I in the hand or arm scheduled for hand or arm surgery, intravenous regional blockade with lidocaine and clonidine (1 μg/kg) showed a relapse rate for clonidine of 10% against 74% in the group receiving only lidocaine [100]. Case studies point to a possible beneficial effect of regional anaesthesia, such as brachial plexus block and epidural anaesthesia [101].

Despite lack of evidence, the task force is of the opinion that operations are preferably postponed until CRPS-I signs are minimal. Preferably, regional anaesthetic techniques such as brachial plexus blockade and epidural anaesthesia should be used (level 4)

*There are indications that stellate blocks and intravenous regional anaesthesia using clonidine (not guanethidine) offer protection (level 3: Reuben et al. (A2))*.

*There are indications that the use of multimodal analgesia offers protection (level 3: Reuben (A2)*.

*There are indications that daily administration of 100 IU of salmon calcitonin s.c. (peri-operatively for four weeks) can prevent a relapse of CRPS-I (level 3: Kissling et al. (B))*.

## Discussion

Besides scientific evidence, other aspects are important in the formulation of guidelines, such as patient perspectives, availability of special techniques or expertise, organisational aspects, social consequences and costs. For the present guidelines these considerations were for a part based on Dutch perspectives. The conclusions based on scientific publications were set into the context of daily practice, and advantages and disadvantages of the various possible policies considered. The final recommendations are the result of the evidence available in combination with these considerations. This procedure followed in the present guideline development provides the opportunity to incorporate the debate between project group members in the formulation of recommendations, in order to make the guideline transparent and bring the recommendations in line with general practice.

Based on the presented evidence based evaluation of CRPS-I literature, in combination with additional considerations with regard to availability of treatment methods, side-effects, cost-benefits and consequences for organisation of care, recommendations endorsed by the participating professional societies were formulated, described additional file 3. In addition to these guidelines the task force is of the opinion that regular consultation between practitioners is desirable in order to provide uniform and clear information to the patient. In line with these observations, the task force advocates that patients should be actively informed about CRPS-I and possible consequences of this complaint, whereby verbal as well as written information should be provided. Although there is no evidence of for specific psychological profile or predisposition for patients with CRPS-I, there may be reasons to carry out further psychological investigation. Possible psychological factors maintaining and/or aggravating the syndrome need to be determined.

A limitation of the guidelines presented in this article is that only articles published up to 2006 were included, and possible relevant findings published after this date couldn't be incorporated in the present guidelines as a consequence of the formal procedure (see method section), involving the approval of participating professional societies. An additional search based on the search string used for these guidelines, revealed 45 additional articles [108-152], possibly providing information that could lead to amendment of this guideline. These articles comprised one retrospective chart review [121], one prospective cohort [120], six case series [117,125,127,128,132,133], 14 clinical trials [109,111,112,119,122,123,126,131,135,138,140,146,147,150], two controlled clinical trials [130,136], 16 RCT's [108,113,114,116,124,129,134,137,139,141,143,145,146,148,149,152], four systematic reviews/meta analyses [110,115,142,144], and one treatment guideline [118]. Interventions evaluated therein were piroxicam [114], gabapentin [126], intrathecal baclofen [146], sympathetic blockade (lumbar, stellate ganglion and intravenous) (n = 5) [122,123,134,143,149], corticosteroids (n = 3) [114,119,135], calcium regulating medication (bisphosphonates, calcitonin) (n = 4) [110,116,138,142], NMDA antagonists (magnesium sulphate, ketamine, memantine) (n = 9) [111,121,125,131,140,146-148,152], free radical scavengers (mannitol, vitamin C) (n = 3) [129,135.137], nitric oxide regulating medication (n = 3) [133,141,151], spinal cord stimulation (n = 5) [112,115,120,128,132], regional anaesthesia (n = 2) [113,117], physiotherapy and rehabilitation medicine (physiotherapy, mirror therapy, manual lymph drainage, vibratory stimulation, functional restoration, sensorimotor retuning, behavioural therapy, occlusional splints) (n = 11) [108,109,124,126,127,130,136,139,144,145,150]. Fifteen studies evaluated a combination of interventions, and four controlled studies used an active control. Two studies addressed primary prevention of CRPS I [117,129]. These studies will have to be evaluated in the next formal adaptation of these guidelines. In addition, since the publication of these guidelines information provided in two studies included in these guidelines [100,101] has been retracted due to scientific misconduct of the author. Recommendations based on these data (i.e. secondary prevention using pre-, per- and postoperative pain control and regional blockades with clonidine) therefore have to be regarded with caution.

Based on the identified literature and the extent of evidence found therein for therapeutic interventions for CRPS-I, we can conclude that further research is needed into each of the modalities discussed in these guidelines. This includes specifically treatment approaches recommended (or not advised) in these guidelines based on expert opinion, such as the use of botulin toxin and tricyclic antidepressants. Scientific data is also lacking with respect to treatment-related aspects, such as the role of the multidisciplinary approach, problems relating to work and communication with the patient and his or her family and close friends.

The project group considers that particular attention needs to be paid to further development of the diagnostic process. This development must be accompanied by research into possible underlying pathophysiological mechanisms (such as genetic factors) associated with CRPS-I, with particular attention being paid to possible sub-groups of the condition related to these underlying mechanisms.

With regard to drug treatment, further investigation is needed into the efficacy of pain medication and the percutaneous sympathetic blockade. More research is also needed into the use of drugs and invasive treatment with children suffering from CRPS-I.

In terms of paramedical treatment, the emphasis must be placed on the difference between pain contingent and a time contingent approach. Research is needed into the effects of various interventions on more long-standing (chronic) CRPS-I and into a multidisciplinary approach to CRPS-I.

## Conclusions

For pain treatment, the WHO analgesic ladder is advised with the exception of strong opioids. For neuropathic pain, anticonvulsants and tricyclic antidepressants may be considered. For inflammatory symptoms, free-radical scavengers (dimethylsulphoxide or acetylcysteine) are advised. To promote peripheral blood flow, vasodilatory medication may be considered. Percutaneous sympathetic blockades may be used to increase blood flow in case vasodilatory medication has insufficient effect. To decrease functional limitations, standardised physiotherapy and occupational therapy are advised. To prevent the occurrence of CRPS-I after wrist fractures, vitamin C is recommended. Adequate perioperative analgesia, limitation of operating time, limited use of tourniquet, and use of regional anaesthetic techniques are recommended for secondary prevention of CRPS-I.

Based on the literature identified and the extent of evidence found for therapeutic interventions for CRPS-I, we conclude that further research is needed into each of the therapeutic modalities discussed in the guidelines.

## Conflicts of interests

The authors declare that they have no competing interests.

## Authors' contributions

All authors have read and approved the final manuscript.

RSGMP is the main author of this manuscript. He participated in establishing the guidelines presented in this manuscript, and contributed to assessment of literature and drawing up text on which these guidelines were based.

JHBG is co-author of this manuscript, and chairman of the guideline development task force. He participated in establishing the guidelines presented in this manuscript, and contributed to assessment of literature and drawing up text on which these guidelines were based.

PEZ, PUD, ILTH, WWAZ and CJGMR are co-authors of this manuscript, and participated as section chairman of the task force for sections of the guidelines. They participated in establishing the guidelines presented in this manuscript, and contributed to assessment of literature and drawing up text on which these guidelines were based.

## Pre-publication history

The pre-publication history for this paper can be accessed here:

http://www.biomedcentral.com/1471-2377/10/20/prepub

## Supplementary Material

Additional file 1 **Search strategy used to identify studies on CRPS**. This file contains the search strings used for literature retrieval for the present guidelines. Click here for file

Additional file 2 **Practical algorithm**. This file contains a practical treatment algorithm based on the recommendations described in these guidelines. Click here for file

Additional file 3 **Recommendations and additional considerations**. This table contains the final recommendations endorsed by the professional societies participating in the guideline development, and the additional considerations related to these recommendations. Click here for file
