# Supplementary material for: Evidence based guidelines for complex regional pain syndrome type 1
Source: BMC Neurol. 2010 Mar 31;10:20. doi: 10.1186/1471-2377-10-20 (PMC2861029; doi:10.1186/1471-2377-10-20)
Supplement: Additional file 1 — Search strategy used to identify studies on CRPS. This file contains the search strings used for literature retrieval for the present guidelines. [file 1471-2377-10-20-S1.DOC]

**Search strategy used to identify studies on CRPS I.**

Conducted in June 2005

###### **PubMed**

(“Complex Regional Pain syndromes”[MeSH] OR “Reflex Sympathetic Dystrophy”[MeSH] OR complex regional pain syndrome [tiab] OR CRPS [tiab] OR reflex sympathetic dystrophy [tiab] OR RSD [tiab] OR Sudeck* [tiab] OR algodystrophy [tiab] OR posttraumatic dystrophy [tiab])

AND

(“Dutch”[LA] OR “English”[LA] OR “French”[LA] OR “German”[LA] OR “Italian”[LA])

AND

(“Meta-analysis” [PT]) OR “Randomized-Controlled-Trial” [PT] OR “Controlled-Clinical-trial” [PT] OR “Clinical Trial” [PT] OR “Review”[PT] OR systematic overview* [tiab] OR systematic review* [tiab])

NOT

(animals [MeSH])

AND

(“Drug Therapy” [MeSH] OR “Therapeutic Uses” [MeSH] OR “Therapeutics” [MeSH])

**Embase**

(‘complex regional pain syndrome type I’/exp OR complex regional pain syndrome;ti,ab OR reflex sympathetic dystrophy:ti,ab OR RSD:ti,ab OR CRPS:ti,ab)

AND

(‘therapy’/exp)

AND

(‘human’/exp)

AND

([comparative study]/lim OR [controlled study]/lim OR [randomization]/lim OR [meta analysis]/ lim OR [review]/lim OR experimental:ti,ab)

AND

[dutch]/lim OR [English]/lim OR [French]/lim or [german]/lim OR [Italian]/lim

CINAHL

((MH "Complex Regional Pain Syndromes+") or (MM "Reflex Sympathetic Dystrophy")) and ((MH "Concurrent Prospective Studies") or (MH "Experimental Studies") or (MH "Multicenter Studies") or (MH "Pilot Studies") or (MH "Prospective Studies") or (MH "Quantitative Studies") or (MH "Nonconcurrent Prospective Studies") or (MH "Quasi-Experimental Studies") or (MH "Case Control Studies") or (MH "Comparative Studies") or (MH "Double-Blind Studies") or (MH "Single-Blind Studies") or (MH "Triple-Blind Studies")) not ((MH "Animal Studies")) and (LA English or LA Dutch or LA German or LA French or LA Italian)

**PsychINFO**

(reflex sympathetic dystrophy)

Limited to:

Methodology is ME=(followup study) or ME=(literature review) or ME=(longitudinal study) or ME=(meta analysis) or ME=(prospective study) or ME=(quantitative study) or ME=(retrospective study) or ME=(systematic review) or ME=(treatment outcome/clinical trial); Language is Dutch or English or French or German or Italian; Publication Type is PT=(journal article); Population is human
