# Supplementary material for: Evidence based guidelines for complex regional pain syndrome type 1
Source: BMC Neurol. 2010 Mar 31;10:20. doi: 10.1186/1471-2377-10-20 (PMC2861029; doi:10.1186/1471-2377-10-20)
Supplement: Additional file 2 — Practical algorithm. This file contains a practical treatment algorithm based on the recommendations described in these guidelines. [file 1471-2377-10-20-S2.DOC]

Practical algorithm

*Trauma, operation,*

*initiating event*

*Determining CRPS-I diagnosis and clinical features*

**Treatment:**

*Paramedical:*

Standardized physical therapy

Standardized occupational therapy

*Medicinal:*

- Pain medication according to WHO pain ladder (up to step 2)
- DMSO 50%/n-acetylcysteine

*Communication and education*:

- oral and written
- involve next of kin
- involve patient organization

*Primary prevention:*

- with wrist fractures Vitamin C

*Secondary prevention (with present or previous CRPS-I):*

- postpone surgery until CRPS-I features have disappeared
- minimize duration of surgery and use of tourniquet
- use adequate pre-, and peroperative analgesia

Consider:

- perioperative ganglion stellatum blockade or regional i.v. anaesthesia with clonidine
- anaesthesia with sympathicolytic effect
- perioperative calcitonine

In case of allodynia/hyperalgesia:

- Gabapentin
- Carbamazepin
- Amytriptilin/nortryptilin

In case insufficient:

- intrathecal baclofen in specialized clinic

In case of dystonia, myoclonia or muscle spasms:

- oral baclofen, diazepam or clonazepam.

In case of cold CRPS I:

- vasodilatory medication

In case of discrepancy between objective complaints and pain behaviour, stagnation of treatment, extensive suffering by patient

- consult psychologist

In case insufficient:

- percutaneous sympathetic blockade

*Other interventions:*

In case of insufficient effect of other therapy, consider:

- spinal cord stimulation in specialized clinic

In case of recurring infections, consider:

- amputation in specialized clinic

*Treatment of children with CRPS I:*

- besides medicinal treatment physical therapy and/or occupational therapy.
- if required, psychosocial guidance by child/youth psychologist
