# Supplementary material for: Evidence based guidelines for complex regional pain syndrome type 1
Source: BMC Neurol. 2010 Mar 31;10:20. doi: 10.1186/1471-2377-10-20 (PMC2861029; doi:10.1186/1471-2377-10-20)
Supplement: Additional file 3 — Recommendations and additional considerations. This table contains the final recommendations endorsed by the professional societies participating in the guideline development, and the additional considerations related to these recommendations. [file 1471-2377-10-20-S3.DOC]

Recommendations and additional considerations.

| Treatment | Evidence level | Considerations | Recommendations |
| --- | --- | --- | --- |
| **_{0>_Medicamenteuze en invasieve behandeling_<}75{>_Drug treatment and invasive treatment_<0}_** | | |  |
| Pain medication  - paracetamol  - NSAID’s  - tamadol  - opioids  - ketamine | III-IV | Use of paracetamol has a low threshold of administration and a minor side-effects profile. NSAIDs are often associated with side-effects (i.e. gastrointestinal, renal, circulatory, central nervous system, and cardiac function). _{0>_Veel bijwerkingen worden beschreven bij de verschillende zwak en sterk werkende opioïden.210_<}0{>_Many side effects have been described for weak and strong-acting opioids._<0}_ S_{0>_Hoewel specifieke informatie met betrekking tot zwak en sterk werkende opioïden voor CRPS-I nagenoeg ontbreekt, wordt in systematische reviews over de toepassing bij neuropathische pijn tramadol als effectief aangemerkt.213 Tevens worden positieve kortetermijneffecten voor sterk werkende opioïden gemeld voor neuropathische en spierskeletpijn.214 Over langetermijneffecten van opioïden, alsmede problemen over tolerantie en verslaving kan vooralsnog geen eenduidige uitspraak worden gedaan.214_<}0{>S_ystematic reviews for neuropathic pain have found tramadol to be effective [14]. Long-term effects of opioids or problems associated with tolerance and addiction for CRPS-I are unkown [15]. Use of ketamine should be limited to the clinical setting. | - _{0>_• Bij CRPS-I met pijnklachten is een subanesthetische dosis ketamine te overwegen._<}0{>_A sub-anaesthetic dose of ketamine can be considered for patients with CRPS-I who are experiencing pain symptoms._<0}_ _{0>_De werkgroep is van mening dat toediening van pijnmedicatie volgens de WHO-pijnladder tot en met stap 2 aan te bevelen is._<}0{>_The project group is of the opinion that pain medication should be administered in accordance with the WHO pain ladder up to and including step 2._<0}_ _{0>_Sterke opioïden dienen bij deze patiëntengroep niet te worden toegepast._<}0{>_Strong opioids should not be administered to this patient group. - The project group also recommends that further research should be carried out into the specific effect of pain medication on CRPS-I._<0}_ |
| Co-analgesics  - gabapentine  - carbamazepin  - pregabaline  - amitryptiline  - nortryptiline | II-IV | Pain associated with CRPS-I may be neuropathic in nature._<0}_ _{0>_Gezien de ervaringen uit andere onderzoeken valt het gebruik van antidepressiva bij continue neuropathische pijn bij CRPS-I te overwegen.<}89{>TheThe use of antidepressants, anticonvulsants to treat neuropathic pain in CRPS-I should be considered. Possible side effect profiles of these medications (for instance dizziness, sleepiness and fatigue) should be taken into consideration. | - _{0>_• Bij patiënten met het CRPS-I valt het gebruik van gabapentine te overwegen._<}0{>_Administration of gabapentin can be considered for patients with CRPS-I._<0}_ _{0>_Indien een proefperiode van acht weken geen duidelijke pijnklachtenreductie of vermindering van de allodynie en hyperesthesie oplevert, dient het medicijn te worden gestaakt._<}0{>_This should be discontinued if no clear reduction in pain symptoms, allodynia or hyperaesthesia occurs within an eight-week trial period. - _{0>_• Indien bij CRPS-I aanvalsgewijze neuropathische pijn op de voorgrond staat, kan een proefbehandeling met carbamazepine, pregabaline of andere anti-epileptica worden overwogen._<}0{>_A trial course of carbamazepine, pregabalin or other anti-epileptic drugs can be considered for patients with CRPS-I suffering significant attacks of neuropathic pain._<0}_ - A trial course of amitriptyline or nortriptyline can be considered for patients with CRPS-I who are suffering from continuous neuropathic pain._<0}_ |
| Capsaicin | IV |  | - Capsaicin has no place in the treatment of CRPS-I. |
| Free radical Scavengers  - DMSO  - N-acetylcystein | II-IV | In light of a possible inflammatory pathofysiological mechanism of CRPS-I, treatment with free radical scavengers has been proposed, with positive results reported in different studies. This treatment method is predominantly used in The Netherlands. | - _{0>_• Bij patiënten die minder dan een jaar CRPS-I hebben, is DMSO (dimethylsulfoxide)-crème 0% dd (lokaal op de huid te appliceren) gedurende drie maanden aan te bevelen._<}0{>_A three-month course of 50% DMSO (dimethylsulphoxide) cream five times a day (for local application on skin) is recommended for patients who have had CRPS-I for less than a year. - _{0>_• Bij patiënten die langer dan een jaar CRPS-I hebben, kan een proefbehandeling met DMSO-crème dd lokaal gedurende een maand worden overwogen._<}0{>_A one-month trial course of DMSO applied locally can be considered for patients who have had CRPS-I for more than a year._<0}_ _{0>_Bestaat er een gunstig effect, dan kan de behandeling gedurende drie maanden worden gecontinueerd._<}0{>_If the results are favourable, the treatment can be continued for three months._<0}_ - A three-month course of 600 mg of N-acetylcystein 3 times a day can be considered for patients with CRPS-I who have a primary cold skin temperature._<0}_ |
| Muscle relaxants  - baclofen (oral)  - diazepam  - clonazepam  - botulin toxin  - baclofen (IT) | III | _{0>_Hoewel anticholinergica, carbamazepine en magnesiumzouten ook worden gebruikt bij de behandeling van dystonie of spasmen bij patiënten met CRPS-I, behoren deze vormen van medicatie strikt genomen niet tot de groep van spierrelaxantia._<}0{>{0>_Ten aanzien van zowel dystonie als spasmen bestaat er geen gecontroleerd onderzoek bij de behandeling van dystonie/spasmen bij CRPS-I. Twee van de hierboven genoemde descriptieve onderzoeken melden dat anticholinergica nimmer (blijvend) effect hebben laten zien.237,238 Bij gebruik van diazepam of clonazepam dient de voorschrijver alert te zijn op mogelijk verslavingsgevaar._<}0{>_Descriptive studies show that anticholinergics do not lead to (lasting) effects [29,30]. Physicians using diazepam or clonazepam must be alert to the possible addiction risk. The main side-effects of the screening process and continuous administration of intrathecal baclofen are post-puncture headache, diminished consciousness and urine retention [33]. | - The members of the project group recommend that CRPS-I patients suffering from dystonia, myoclonias or muscle spasms should be started on 1) oral baclofen according to the standard dose increase pattern, 2) diazepam or clonazepam, which should be slowly titrated in the light of the effect and side-effects. - _{0>_• De werkgroep is van mening dat er geen plaats is voor de behandeling met botulinetoxine bij CRPS-I-patiënten met dystonie._<}0{>_The project group considers that botulin toxin has no place in the treatment of CRPS-I-patients with dystonia._<0}_ - _{0>_• Er is geen plaats voor behandeling met intrathecale baclofen (ITB) bij patiënten met CRPS-I. Intrathecale baclofen kan uitsluitend worden overwogen bij patiënten met CRPS-I indien dystonie op de voorgrond staat en conventionele therapie geen effect heeft gehad._<}0{>_Intrathecal baclofen has no place in the treatment of patients with CRPS-I. Intrathecal baclofen can only be considered for patients with CRPS-I if dystonia is a major problem and conventional therapy has proven ineffective._<0}_ _{0>_De behandeling dient te worden uitgevoerd in onderzoeksverband._<}0{>_This treatment must be administered in the context of a trial._<0}_ |
| Corticosteroids | III | Some studies of limited quality indicate that corticosteroids have a beneficial effect._<0}_ _{0>_Gezien de bijwerkingen adviseert de werkgroep corticosteroïden niet routinematig te gebruiken._<}0{>_ | - Routine administration of corticosteroids has no place in the treatment of CRPS-I patients._<0}_ |
| Calcium regulating medication  - calcitonin  - bis-phosphonates | I | Experience with calcitonin and bisphosphonates for CRPS-I patients in The Netherlands is limited. Intravenous bisphosphonates cause few side effects, but dosage, frequency and duration of use are unclear._<0}_ _{0>_Alendronaat dagelijks 40 mg gedurende acht weken kan worden overwogen, vooral bij een verhoogd botmetabolisme.<}0{>Treatment Treatment with 40 mg of alendronate/day for eight weeks may be considered, especially for patients with elevated bone metabolism. | - The project group is of the opinion that in view of the conflicting results of research, it is impossible to give any clear advice about the use of calcitonin in patients with CRPS-I. - As there is little experience with the use of bisphosphonates in patients with CRPS-I, it is currently advised that these drugs should only be considered in the context of a trial. |
| Calcium channel blockers | III |  | - A calcium channel blocker can be prescribed for patients with a cold CRPS-I._<0}_ _{0>_Een week na behandeling moet het effect hiervan worden geëvalueerd._<}0{>_The effect must be assessed a week after administration._<0}_ _{0>_Bij uitblijven van effect moet deze therapie worden gestaakt._<}0{>_The drug must be discontinued if it has no effect. |
|  | | |  |
| Sympathetic block  - Intravenous  - Percutaneous | I-II | The task force is of the opinion that percutaneous sympathetic blockade may be helpful in improving the circulation in patients with cold CRPS-I. _{0>_Hoewel het logisch lijkt en is gesuggereerd dat operatieve sympathectomie vooral is geïndiceerd bij patiënten met bevestigde ‘sympathicusafhankelijke pijn’,29 zou volgens anderen het behandelresultaat hier niet mee zijn gecorreleerd.296 Na sympathectomie voor neuropathische pijn treedt compensatoire hyperhidrose op bij 18% en neuropathische complicaties bij 25% van de patiënten.298_<}0{>_ | - Intravenous sympathetic blockade has no place in the treatment of patients with CRPS-I. - _{0>_• Bij patiënten met een koude CRPS-I die niet voldoende reageren op vaatverwijdende medicatie, kan een percutane sympathicusblokkade met lokale anesthetica worden overwogen._<}0{>_Treatment with percutaneous sympathetic blockade using local anaesthetics may be considered for patients with cold CRPS-I who do not respond adequately to vasodilating medication._<0}_ _{0>_• Het routinematig intraveneus toedienen van reserpine, droperidol en atropine wordt niet aanbevolen bij CRPS-I-patiënten._<}0{><0}_ - If a trial blockade has proved successful, definitive sympathetic blockade using radiofrequent lesions, phenol or alcohol can be considered in the context of a study. |
| Surgical sympathectomy | III | Surgical sympathectomy is proposed to be indicated for 'sympathetic-dependent pain' [64], but there is discussion on this issue [63]. Compensatory hyperhidrosis and neuropathic complications are common with this intervention [60] | - _{0>_• Grote terughoudendheid moet worden betracht bij de toepassing van een operatieve sympathectomie voor pijnbestrijding bij CRPS-I. Om effectiviteit en potentiële risico’s in kaart te brengen dient de behandeling in onderzoeksverband plaats te vinden._<}0{>_Extreme caution is necessary when considering surgical sympathectomy for pain control in CRPS-I. The procedure should be conducted in the context of a trial in order to ascertain the efficacy and potential risks._<0}_ |
| Other intravenous treatment:  - ketanserine  - bretylium  - reserpine  - droperidol  - atropine | I | Bretylium is not registered in the Netherlands. Reserpine, droperidol and atropine have insufficient effect. | - _{0>_• Het intraveneus toedienen van 10-20 mg ketanserine kan worden overwogen bij de behandeling van CRPS-I-patiënten._<}0{>_Intravenous administration of 10-20 mg of ketanserine can be considered for the treatment of CRPS-I patients. - Routine administration of reserpine, droperidol and atropine is not recommended for CRPS-I patients._<0}_ |
|  | | |  |
| Spinal cord  stimulation (SCS) | III | Evidence for use of SCS in non-chronic CRPS-I is lacking. SCS studies were performed in carefully selected refractory CRPS-I patients. SCS is more c_{0>_Een kosteneffectiviteitsanalyse toonde dat behandeling van chronisch CRPS-I met ruggenmergstimulatie goedkoper is dan standaardtherapie.288_<}0{>_ost-effectiveness than standard therapy for chronic CRPS-I [102]._<0}_ _{0>_Hoewel levensbedreigende complicaties bij ruggenmergstimulatie zeldzaam zijn, treden complicaties leidend tot reoperatie wel op bij 2 - 0% van de patiënten.289_<}0{>_Life-threatening complications are rare, but complications occur frequently [70]. | - _{0>_• Pijnbestrijding met ruggenmergstimulatie bij nauwkeurig geselecteerde, chronisch CRPS-I-patiënten die niet hebben gereageerd op overige therapieën, is verantwoord._<}0{>_Pain control with spinal cord stimulation is a sound option for carefully selected CRPS-I patients who have not responded to other treatments._<0}_ _{0>_Toepassing van ruggenmergstimulatie bij overige CRPS-I-patiënten dient bij voorkeur plaats te vinden in onderzoeksverband._<}0{>_Spinal cord stimulation should ideally only be administered to other CRPS-I patients in the context of a trial._<0}_ |
| Amputation | III | Amputation of the affected limb cannot be considered for symptom relief in CRPS-I. It should only be considered in cases of potentially life-threatening, untreatable or recurrent infections. | - _{0>_• Amputatie kan bij CRPS-I-patiënten alleen worden overwogen in een gespecialiseerd centrum bij ernstig recidiverende infecties en bij ernstige functiestoornissen om de kwaliteit van leven te verbeteren._<}0{>_Amputation for CRPS-I patients can only be considered in order to improve the quality of life in the case of severe, recurrent infections and severe functional disorders. This intervention should be performed at a specialised centre._<0}_ |
| **Paramedical, rehabilitation medicine and psychological treatment** | | |  |
| Physiotherapy and TENS | II-IV | In the view of the task force, physiotherapy can positively influence the patient's ability to exert control as a part of a pain-focused treatment protocol. _<0}_There are no contraindications for physiotherapy. | - _{0>_• Het is aan te bevelen fysiotherapeutische behandeling, waarbij functioneel herstel centraal staat, in een zo vroeg mogelijke fase van CRPS-I te starten._<}0{>_It is recommended that physiotherapy aimed at restoration of function be started as soon after the onset of CRPS-I as possible._<0}_ - _{0>_• TENS (transcutane elektrische zenuwstimulatie) kan zonder risico als aanvullende therapie worden geprobeerd bij CRPS-I-patiënten._<}0{>_TENS (transcutaneous electrical nerve stimulation) can be tried out without risk in CRPS-I patients as an additional treatment._<0}_ _{0>_Het is alleen zinvol de behandeling te continueren als deze als werkzaam wordt ervaren._<}0{>_It is only sensible to continue with the treatment if it is found to be effective._<0}_ |
| Occupational therapy | III | _{0>_Er zijn geen contra-indicaties voor ergotherapie bekend._<}0{>{0>_Het bevorderen van het functioneel gebruik van de extremiteit binnen de pijngrens en het bevorderen van de zelfstandigheid zijn tevens belangrijke doelen van de ergotherapie.316_<}0{>_Promoting functional limb use within pain limits and promoting independence are aims of occupational therapy [103]. _{0>_Desensitisatieprogramma’s als onderdeel van de ergotherapiebehandeling worden toegepast bij het ‘normaliseren van de sensibiliteit’.312 Deze programma’s, waarin een opbouw van stimuli wordt gegeven, hebben verhoging van de tolerantie van tactiele prikkels tot gevolg.198,31_<}0{>_Desensitisation programmes are used to normalise sensitivity [6,104,105]._<0}_ _<0}{0>_Spalkbehandeling lijkt geïndiceerd als het een onderdeel is van de totale ergotherapiebehandeling en gaat altijd gepaard met draaginstructies._<}0{>{0>_De spalk is bij voorkeur functioneel ondersteunend, maar kan tevens bescherming bieden en gericht zijn op het verminderen van de klinische symptomen._<}0{>_Splints are used to provide functional support, protection and _<0}{0>_Er wordt naar gestreefd in de loop van de tijd het gebruik van de spalk qua duur en frequentie af te bouwen.312 De spalk dient bij voorkeur niet immobiliserend te zijn.198,31 ,316 Dat wil zeggen dat de spalk bij voorkeur niet continu wordt gedragen, en dat zo veel mogelijk (bewegingsmogelijkheden van de) gewrichten vrij worden gehouden._<}0{>_ minimising clinical symptoms [6,103,106]. There are no known contraindications for occupational therapy._<0}_ | - The project group recommends that patients with upper-limb CRPS-I be referred for occupational therapy. |
| Psychological treatment | IV | There is no evidence for a specific psychological profile or predisposition for CRPS-I patients. Reasons for further psychological investigation relate to assessment of possible psychological factors maintaining and/or aggravating the syndrome. | - The project group advises that CRPS-I patients should consult a psychologist if the practitioner observes a discrepancy between clinical symptoms and the patient's (pain-related) behaviour, if stagnation in (somatic) treatment occurs, if the burden of suffering caused by the symptoms is great, or if the patient requests this. |
| Multidisciplinary treatment | IV | _{0>_• De behandeling vraagt om betrokkenheid van diverse disciplines._<}0{>_Treatment of CRPS-I may require the involvement of various disciplines due to its multidimensional character._<0}_ R_{0>_De behandeling dient zowel te zijn gericht op stoornissen (pijnbestrijding, herstel van vegetatieve ontregelingen) als op beperkingen voor de patiënt (bijvoorbeeld herstel van hand- of loopfunctie)._<}0{>{0>_• Patiënten met chronisch CRPS-I ervaren problemen op diverse gebieden._<}0{>{0>_Naast het functieverlies en de hiermee gepaard gaande sociale en beroepsmatige problemen is er vaak ook, door de gevolgen van de aandoening, psychosociale problematiek._<}0{><0}{0>_• Regelmatige onderlinge afstemming tussen behandelaars is gewenst, zodat een eenduidige boodschap aan de patiënt kan worden gericht._<}0{>_egular consultation between practitioners is desirable to provide uniform information to the patient. | - _{0>_• Als er diverse behandelaars gelijktijdig bij de behandeling van CRPS-I-patiënten zijn betrokken, is het wenselijk dat er één behandelaar optreedt als ‘case-manager’._<}0{>_Where a number of practitioners are treating a CRPS-I patient at the same time, it is advisable that one of them acts as case manager._<0}_ |
| **Treatment of children with CRPS-I** | | |  |
| Drug and invasive treatment | III | Too little data is available to allow a balanced conclusion with regard to the effects of the different interventions on children with CRPS-I. _{0>_Als medicamenteuze en invasieve behandelingen zoals beschreven in deze richtlijn bij kinderen worden toegepast, dient het bovenstaande in ogenschouw te worden genomen.<}0{>PractitionersParticular attention should be given to medication dosage and to support for the child during the disease process._<0}_ _{0>_Nauwe samenwerking met een kinderarts lijkt in dit kader gerechtvaardigd._<}0{>_Close cooperation with a paediatrician appears justified. _{0>_In het algemeen wordt een scala van verschillende interventies tegelijkertijd of in sequentie toegepast, zoals oefentherapie, massage, contrastbaden en TENS.189-19 ,309,33 ,337 Een psycholoog wordt zeer vaak ingeschakeld (diagnostisch en therapeutisch) bij de behandeling van kinderen met CRPS-I.189-19_<}0{>_Psychologists are often involved in the treatment of children with CRPS-I [85,86,89-92]. _{0>_De behandeling van kinderen met CRPS-I richt zich net als bij volwassenen vooral op de gevolgen van de klachten._<}0{>{0>_Cognitieve gedragstherapie, waarbij relaxatiemethoden kunnen worden gebruikt, is in onderzoek uit de Verenigde Staten effectief gebleken._<}0{>{0>_Bij de behandeling van kinderen is het verstandig de ouders of het hele gezin in de behandeling te betrekken._<}0{>_When treating children it is advisable to include the parents or family._<0}_ | - _{0>_• De werkgroep is van mening dat nader onderzoek noodzakelijk is om de effecten van medicamenteuze en invasieve interventies bij kinderen met CRPS-I in kaart te brengen._<}0{>_The project group is of the opinion that further research is needed to determine the effects of drug treatment and invasive treatment on children with CRPS-I._<0}_ _{0>_Voorzichtigheid is geboden bij het toepassen van in deze richtlijn beschreven behandelingen bij kinderen._<}0{>_Caution is advised when applying the treatments described in these guidelines to children._<0}_ _{0>_Bijzondere aandacht dient hierbij uit te gaan naar dosering en (medische) begeleiding van het kind._<}0{>_Particular attention must be paid to measurement of the dose and giving (medical) support to the child._<0}_ |
| Physical therapy | III |  | - The project group recommends that children with CRPS-I should be given physiotherapy. |
| Occupational therapy | III |  | - The project group advises that occupational therapy should be a component of multidisciplinary treatment for children with CRPS-I. |
| Psychological therapy | II |  | - Psychological diagnosis and treatment of children with CRPS-I should ideally be carried out by a child psychologist. |
| **Primary and secondary prevention of CRPS-I** | | |  |
| Primary prevention  - Vitamin C  - Guanethidine  - Calcitonin | II-III | Vitamin C is available at low cost. Guanethidine is not available in The Netherlands. Perioperative administration of calcitonin has insufficient effect on primary prevention of CRPS-I | - Consideration should be given to prescribing 500 mg of vitamin C to be taken orally for 50 days in order to reduce the risk of CRPS-I in adults who have had a wrist fracture. - Perioperative administration of intravenous guanethidine is not advised for primary prevention of CRPS-I. - Perioperative administration of subcutaneous calcitonin is not advised for primary prevention of CRPS-I. |
| Secondary prevention | III-IV | _{0>_In het algemeen lijkt het verstandig te wachten met opereren in een extremiteit met CRPS-I tot de symptomen en verschijnselen van CRPS-I zijn geminimaliseerd.355-359 Het kan echter ook zijn dat er een factor is die de CRPS-I onderhoudt (‘triggerpoint’) en dat de operatie gericht is op die factor.<}0{>IIt appears sensible to wait until the signs and symptoms of CRPS-I have abated before performing surgery on CRPS-I patients [98-101,107]. Surgery should not be postponed in case the surgery is intended to reduce factors maintaining the CRPS-I [98]. Surgery on cold, oedematous limbs is not advisable [98]. _<0}_ | - Timing of surgery:_<0}_ _{0>_Het verdient aanbeveling te wachten met een chirurgische ingreep aan de (voorheen) aangedane extremiteit totdat de symptomen en verschijnselen van CRPS-I nagenoeg verdwenen zijn._<}0{>_It is recommended that surgery of the (previously) affected limb be postponed until the signs and symptoms of CRPS-I have almost disappeared._<0}_ _{0>_Dit geldt niet indien de operatie tot doel heeft een mogelijk onderhoudende factor voor de CRPS-I weg te nemen._<}0{>_This does not apply to operations intended to eliminate an underlying factor that may be responsible for the CRPS-I. - It is recommended that the duration of the operation and use of tourniquet be minimised._<0}_ - Adequate pre-, per- and postoperative pain control is recommended. - Perioperative blockades of the ganglion stellatum or IV regional blockades using clonidine 1 µg/kg (not guanethidine) can be considered in the case of upper-limb surgery on patients who previously suffered from CRPS-I. - _{0>_• Het gebruik van regionale anesthesie met sympathicolytisch effect (epidurale/spinale analgesie, plexus brachialis-blokkade), al dan niet gecombineerd met algehele anesthesie, kan worden overwogen bij operaties bij patiënten met doorgemaakte CRPS-I._<}0{>_The use of regional anaesthesia with a sympathicolytic effect (epidural/spinal analgesia, plexus brachialis blockade), either alone or in combination with general anaesthesia, can be considered in the case of surgery on patients who previously suffered from CRPS-I._<0}_ - The perioperative use of calcitonin can be considered. |
|  |  |  |  |
